# Supplementary material for: Intramyocardial hemorrhage drives fatty degeneration of infarcted myocardium
Source: Nat Commun. 2022 Oct 27;13:6394. doi: 10.1038/s41467-022-33776-x (PMC9613644; doi:10.1038/s41467-022-33776-x)
Supplement: Supplementary file 1 — Supplementary Information [file 41467_2022_33776_MOESM1_ESM.pdf]

Supplementary Material

Content:

1) List of Abbreviations
2) Supplementary Table 1: Specific targets, associated markers and antibodies used to probe myocardial tissue
3) Supplementary Figure 1: Study Timeline
4) Supplementary Figure 2: Lipomatous metaplasia in the early chronic phase of MI is unique to hemorrhagic infarcts and is observed exclusively at the have confluence of ion and lipid
5) Supplementary Figure 3: Absence of foam cells and lipomatous metaplasia in the early chronic phase of non-hemorrhagic MI is linked to absence of reperfusion hemorrhage in the acute phase of MI.
6) Supplementary Figure 4: Mast cells home to iron-laden regions of scar tissue undergoing lipomatous metaplasia in the early phase of chronic hemorrhagic MI
7) Supplementary Figure 5: Lipomatous metaplasia in the late chronic phase of MI is unique to hemorrhagic Infarcts
8) Supplementary Figure 6: Reperfusion hemorrhage-derived iron deposition carries a risk of lipomatous metaplasia in late chronic phase of post-MI scar.
9) Supplementary Figure 7: Lipomatous metaplasia in the late chronic phase of hemorrhagic MI is unique to scarred regions with iron deposits and lipid remnants.
10) Supplementary Figure 8: Lipomatous metaplasia is not evident in in the late chronic phase of non-hemorrhagic MI.
11) Supplementary Figure 9: Appearance of foam cells in the late chronic phase of hemorrhagic MI is accompanied by highly localized deposition of ceroid lipopigment in iron-laden territories.
12) Supplementary Figure 10: Lipomatous metaplasia of hemorrhagic MI begins in the peri-infarct and border zones of infarcted myocardium and progressively invades the scar core.
13) Supplementary Figure 11: Inability of iron-laden macrophages to switch from M1 to M2 phenotype is coincident with foam cell formation and lipomatous metaplasia in the late chronic phase of hemorrhagic MI.
14) Supplementary Figure 12: Macrophages found in non-hemorrhagic MI territories lack iron and lipid globules.

1) List of Abbreviations:

ε_c	circumferential strain
[Fe]	concentration of iron
AMI	acute myocardial infarction
BL	baseline
BW	bandwidth
CC3	cleaved-caspase 3
CD36	cell surface glycoprotein found on foam cells
CD163	cell surface hemoglobin-haptoglobin receptor
CHF	chronic heart failure
CMR	cardiac MRI
D3	3 days after MI
DFP	deferiprone
DFP+	treated with deferiprone
DFP-	not-treated with deferiprone
DIC	differential interference contrast
EDS	energy dispersive x-ray spectroscopy
EMT	elastin Masson's trichrome
E06	monoclonal antibody (binds to oxidized phospholipids)
ESV	end systolic volume
GLUT-1	glucose transporter 1
hMI	hemorrhagic myocardial infarction
H&E	hematoxylin and eosin
$I:R_{WT}$	infarct-to-remote wall thickness
IL-1β	interleukin 1 beta
IHC	immunohistochemical
IMH	intramyocardial hemorrhage
IMH+	infarctions with intramyocardial hemorrhage
IMH-	infarctions without intramyocardial hemorrhage
LAD	left anterior descending coronary artery
LGE	late-gadolinium-enhancement
LM	lipomatous metaplasia
LV	left ventricle
LVEF	left ventricular ejection fraction
MΦ	unpolarized macrophage
mGRE	multi-gradient echo
MMP-9	matrix metalloproteinase 9
M1	pro-inflammatory macrophage

M2	anti-inflammatory macrophage
M6	6 months after MI
MAC387	monoclonal antibody 387 (binds to new macrophages)
MI	myocardial infarction
MRI	magnetic resonance imaging
ORO	oil-red-O
oxLDL	oxidized low-density lipoprotein
PB	Prussian blue
PCI	percutaneous coronary intervention
PDFF	proton-density corrected fat fraction
R2*	1/T2*
T2*	time constant of free-induction signal decay of MRI signal
TB	toluidine blue
TE	echo time
TEM	transmission electron microscopy
TNF-α	tumor necrosis factor α
TTC	triphenyl tetrazolium chloride
TR	repetition time
Wk8	8 weeks after MI

2) **Supplementary Table 1**: Specific targets, associated markers and antibodies used to probe myocardial tissue

**Target**	**Specific Marker**	**Antibody**	**Dilution/amounts used**
Newly recruited macrophages	MAC387	Abcam ab22506	1 μg/ml
Macrophage scavenger receptor	CD163	Bioss, bs-2527R	1:200
Proinflammatory cytokine	TNF-α	Abcam, ab6671	1:200
Matrix degrading enzyme	MMP-9	Abcam, ab38898	1:200
Proinflammatory cytokine	IL-1β	Abcam, ab34837	1:200
Glucose transporter	GLUT-1	Biorbyt LLC, orb312259	1:200
Oxidized Phospholipids	E06	Avanti Polar Lipids, 330001S	1:100
Foam Cells	CD36	Sigma-Aldrich, AV48129	4 μg/ml
Apoptosis	Cleaved Caspase-3	Cell Signaling, Asp175 (5A1)	1:2000

3) **Supplementary Figure 1**

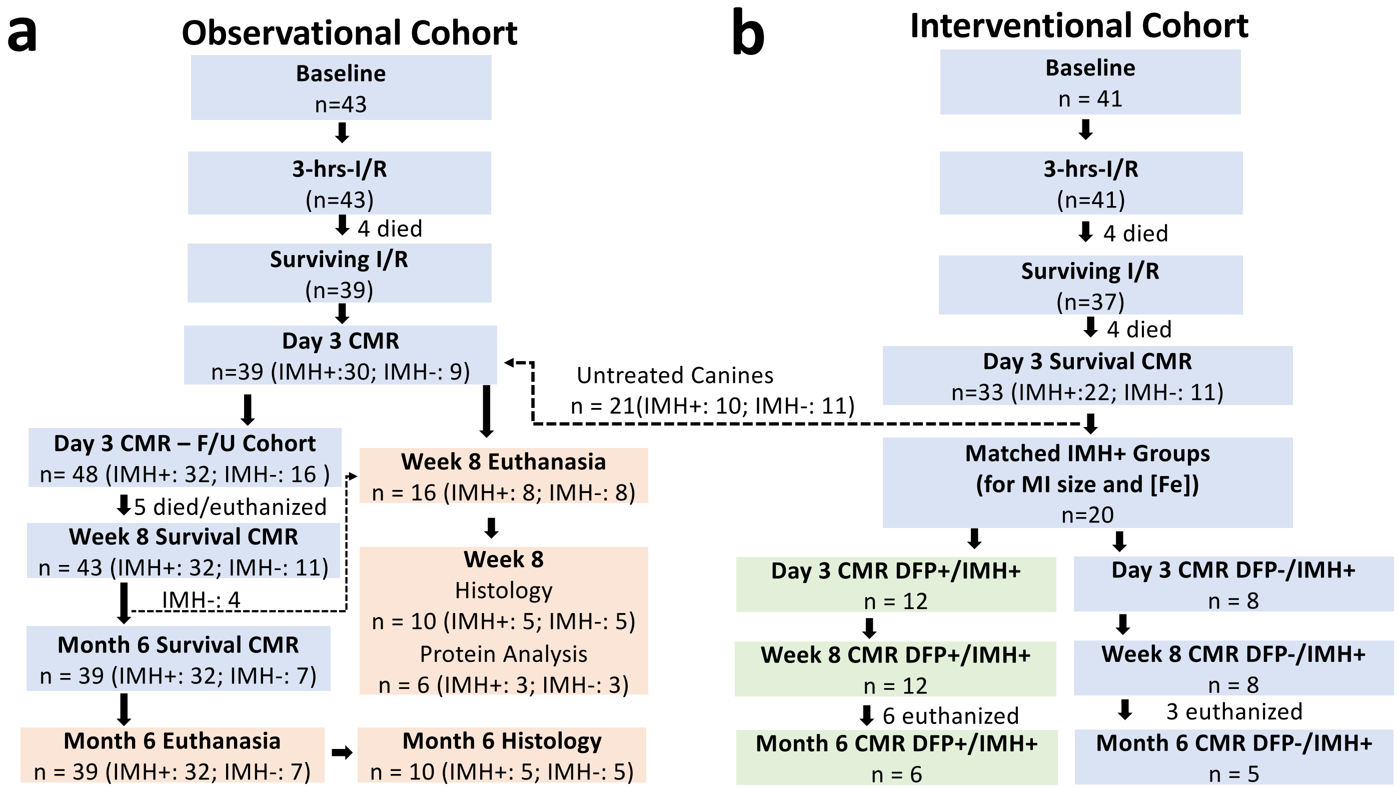

Supplementary Figure 1. Study Timeline and Animal Numbers. The flow charts provide details on the animal groups, time points of CMR, euthanasia and histology. The details related to the observational studies are shown in (**a**) and those related to the interventional studies are shown in (**b**). Note that in (**b**), animal numbers are narrowed down from n = 41 to n = 20 to ensure that animals from DFP+/IMH+ and DFP-/IMH+ groups were matched to MI size and iron content at Day 3 (i.e. prior to treatment)). Whenever possible (dotted lines), untreated animals from the Interventional cohort were shared with Observational cohort to ensure optimal use of animals.

4) **Supplementary Figure 2**

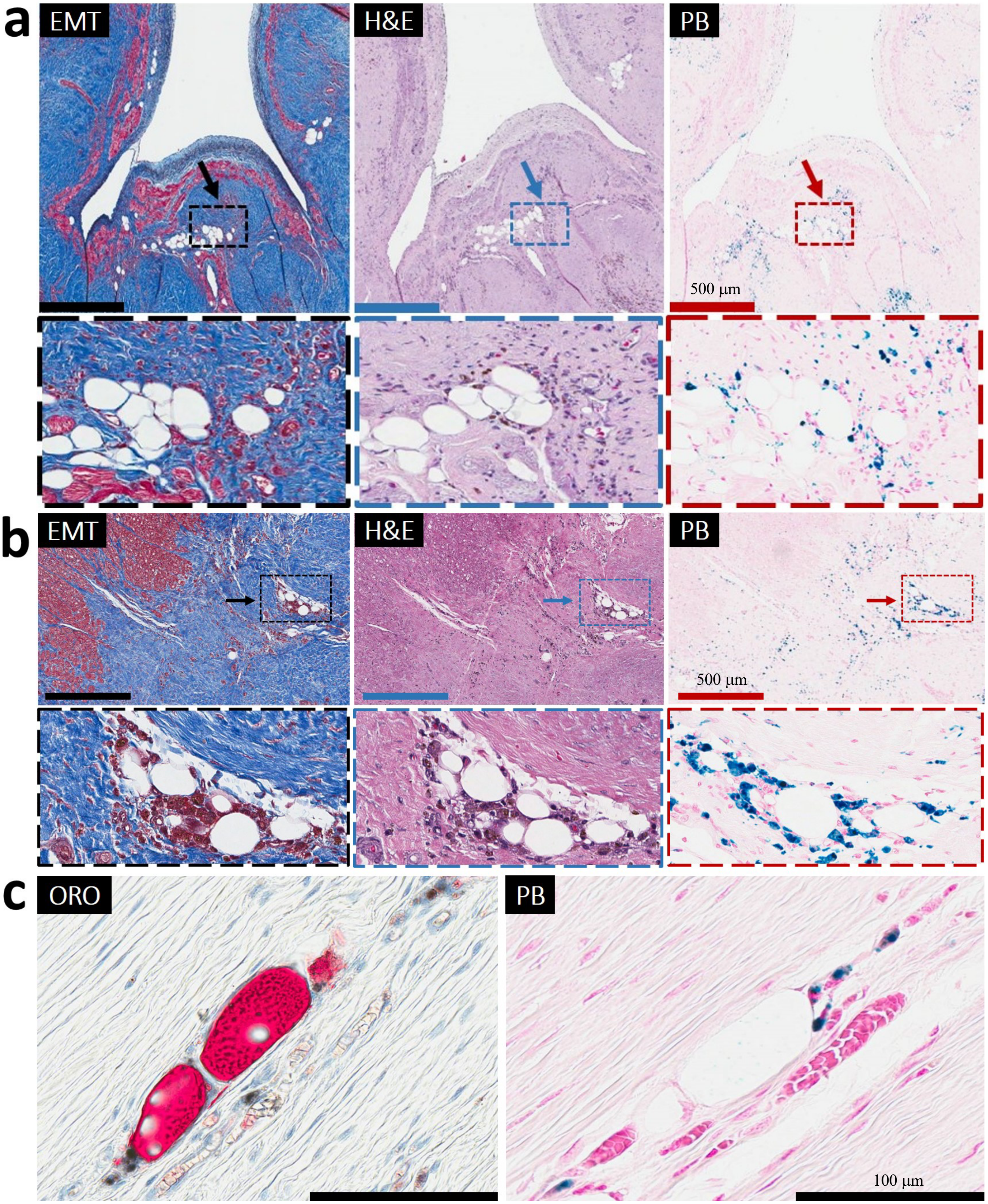

Supplementary Figure 2. Lipomatous metaplasia in the early chronic phase of MI is unique to hemorrhagic infarcts and is observed exclusively at the confluence of iron and lipid. Serial *paraffin* sections from an 8-week-old hemorrhagic MI stained with elastin-modified Masson's trichrome (EMT), H&E and Prussian Blue (PB) from peri-infarct zone of sub-endocardium and midmyocardium is presented in panels **a** and **b**, respectively (zoomed-in sections of panels **a** and **b** presented "in square"/dotted line boxes/rectangles). Individual foam cells were exclusively observed in the peri-infarct and border zones of hemorrhagic MIs and exclusively co-localized with residual iron deposits. Serial *frozen* sections from an 8-week old hemorrhagic MI stained with Oil-Red-O (ORO) and PB stains are presented in panel **c**. As evident in panel **c**, foam cells were observed only at the confluence of iron (PB stained regions) and lipid deposits (ORO regions). Moreover, panel **c** supports the notion that even small traces of iron deposits from reperfusion hemorrhage, if in contact with lipid remnants from necrotic myocardium, carry a risk for LM. The number of samples per timepoint/animal group used is depicted in Supplementary Fig. 1. Scale bar in panels **a** and **b** equals 500 μm while in panels **c** equals 100 μm.

5) **Supplementary Figure 3**

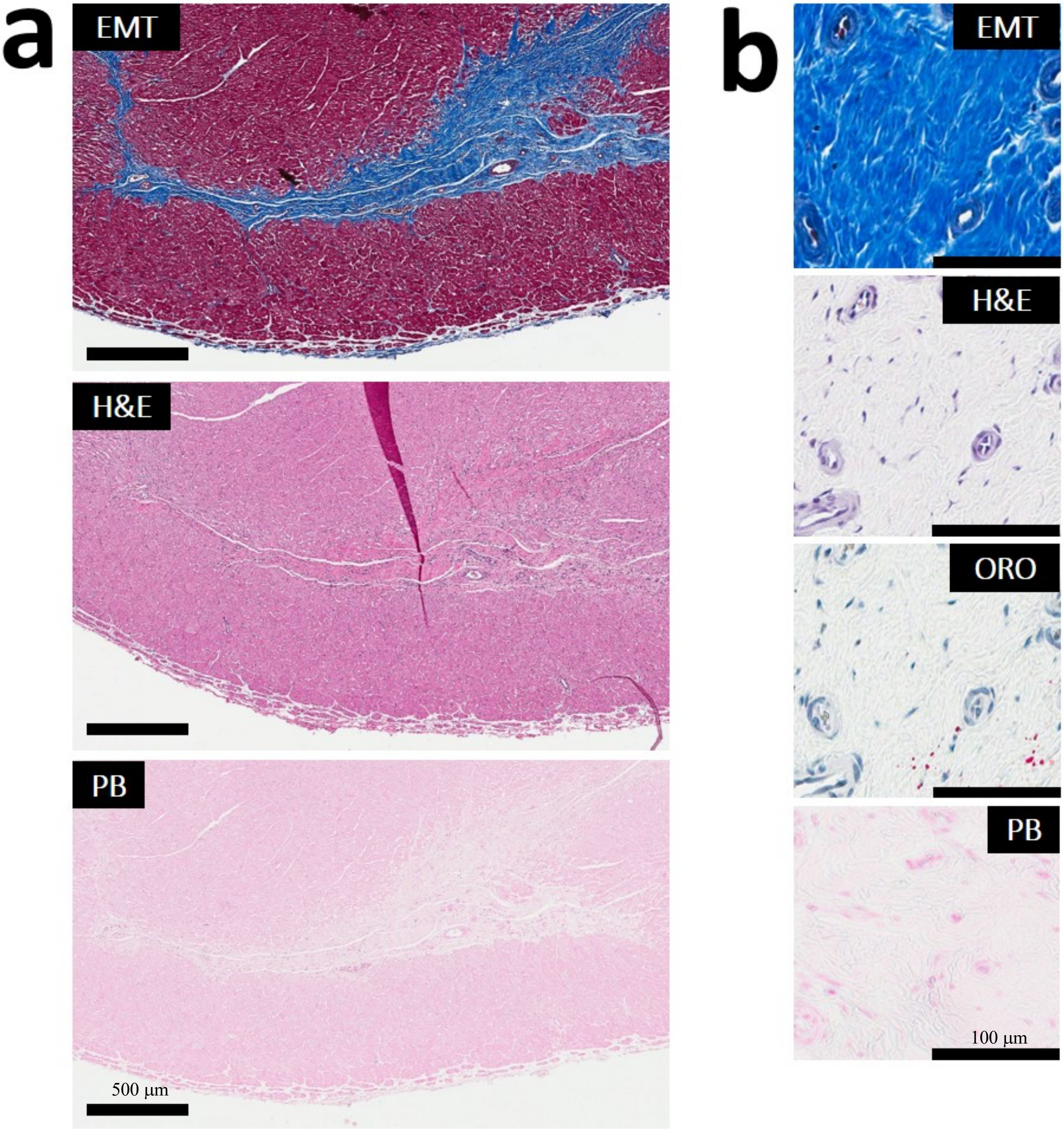

Supplementary Figure 3. Foam cells and lipomatous metaplasia in the early chronic phase are absent in non-hemorrhagic MI. Serial *paraffin* sections from an 8-week-old non-hemorrhagic MI stained with elastin-modified Masson's trichrome (EMT), H&E and Prussian Blue (PB) are presented in panel **a**. Non-hemorrhagic MIs were consistently negative for iron and foam cells in the early phase of chronic MI. Given that these foam cell-negative scars consistently exhibited traces of ORO-stained lipid remnants in contiguous *frozen* sections (panel **b**), our data suggests a critical role for iron deposits in triggering lipid peroxidation, foam cell formation, LM and adverse ventricular remodeling. The number of samples per timepoint/animal group used is depicted in Supplementary Fig. 1. Scale bar in panel **a** equals 500 μm while in panel **b** equals 100 μm.

6) **Supplementary Figure 4**

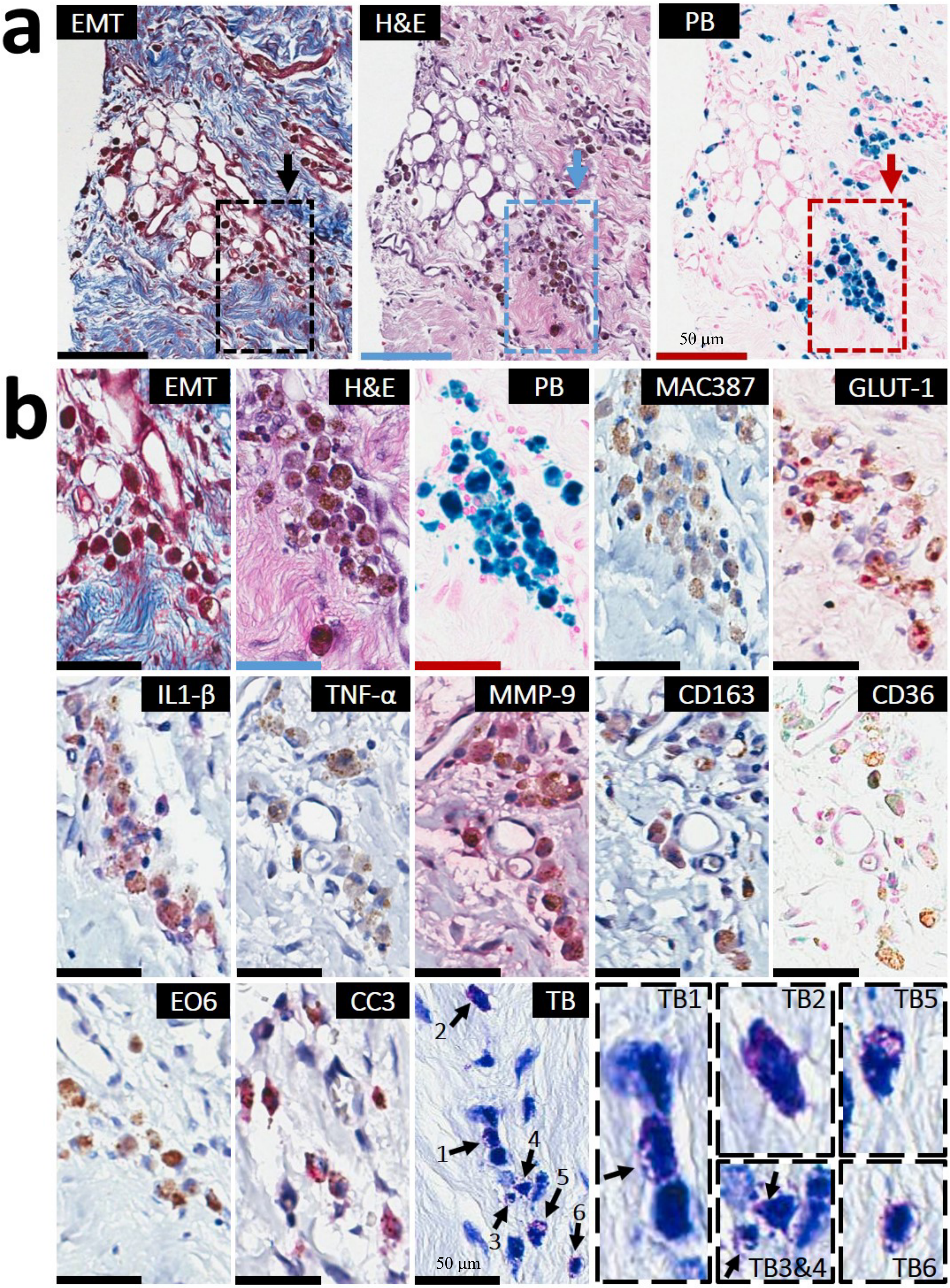

Supplementary Figure 4. Mast cells home to iron-laden regions of scar tissue undergoing lipomatous metaplasia in the early chronic phase of hemorrhagic MI. Representative serial histology sections from 8-week-old hemorrhagic MI were stained with Panel **a**: elastin-modified Masson's trichrome (EMT) stain, H&E, Prussian Blue (PB), as well as Panel **b**: Toluidine Blue (TB), anti-Cleaved Caspase 3 (CC3), anti-MAC387, anti-E06, anti-IL-1β, anti-TNF-α, anti-MMP-9, anti-CD163, anti-CD36, and anti-GLUT-1 antibodies. Note the extensive co-localization of iron deposits (PB, blue staining) and foam cells. Positive immunohistochemical (IHC) staining with anti-CC3 antibody confirmed the ongoing apoptosis of iron-laden macrophage-derived foam cells (red stain; arrows). IHC staining with MAC387 antibody (brown staining) indicates that new macrophages are perpetually recruited to the regions with apoptotic iron-laden macrophage-derived ceroid-rich foam cells. Glycolytic M1 macrophage phenotype in macrophages undergoing foam cell transformation was demonstrated by intense immunoreactivity for GLUT-1 and other proinflammatory macrophage markers (IL1-β, TNF-α and MMP-9; all stained red); which indicates that iron-laden macrophages in the ceroid-rich regions preferentially polarize to pro-inflammatory M1 phenotype. CD163-positive staining (pink stain) in iron-rich regions undergoing LM indicates perpetual iron-induced macrophage induction and iron-laden macrophage-derived foam-cell transformation. Intense staining with CD36 antibody confirmed the presence of foam cells. Note also the extensive co-localization of E06-stained oxidized phospholipids (E06, brown staining) with foam cells in iron-laden macrophage-rich regions. Positive IHC staining with anti-CC3 antibody confirmed the ongoing apoptosis of iron-laden macrophage-derived foam cells (red stain; arrows). Note that iron- and iron-laden macrophage-rich zones in the vicinity of scar regions undergoing LM exhibited increased homing and degranulation of mast cells as evident by TB staining. Note the individual degranulated mast cells in TB1-6. The number of samples per timepoint/animal group used is depicted in Supplementary Fig. 1. Scale bar equals 50 μm.

7) **Supplementary Figure 5**

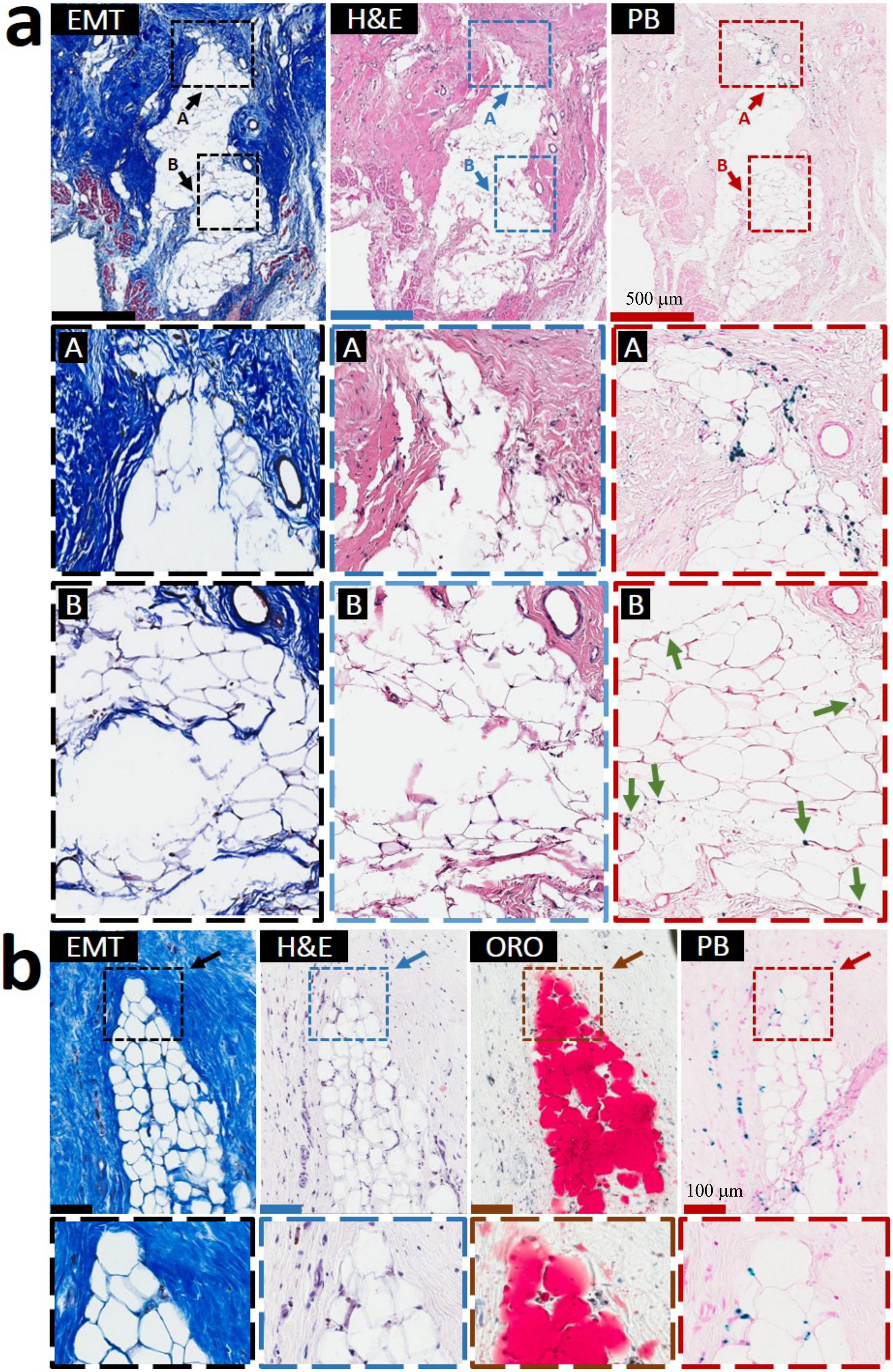

Supplementary Figure 5. Lipomatous metaplasia in the late chronic phase of MI is unique to hemorrhagic Infarcts. Serial *paraffin* sections from an 6-month-old hemorrhagic MI stained with elastin-modified Masson's trichrome (EMT), H&E and Prussian Blue (PB) stains are presented in Panel **a**. Larger fat depots typically penetrated scar tissue at its internal core (Zone **A**) were observed. Notably, these larger foam cell clusters typically colocalized with iron deposits along the fat depot periphery while the core of the growing adipose tissue contained traces of iron deposits (Zone **B**, arrows). Serial *frozen* sections from a dog with 6-month-old hemorrhagic MI stained with H&E, EMT, Oil-Red-O (ORO) and PB stains are presented in Panel **b**. Note the extensive colocalization of iron deposits and foam cells in the fat depot penetrating the internal core of hemorrhagic scar. Additional examples of LM in the peripheral zone of the *sub-endocardium* and *midmyocardium* and at the border zone of the MI territories and its relation to iron and foam cells in 6-month-old scars are shown in Supplementary Fig. 6 and 7. For 6-month-old non-hemorrhagic MI scenario, refer to Supplementary Fig. 8. The number of samples per timepoint/animal group used is depicted in Supplementary Fig. 1. Scale bar of images in panel **a** is 500 μm, while those in panels **b** are 100 μm.

8) **Supplementary Figure 6**

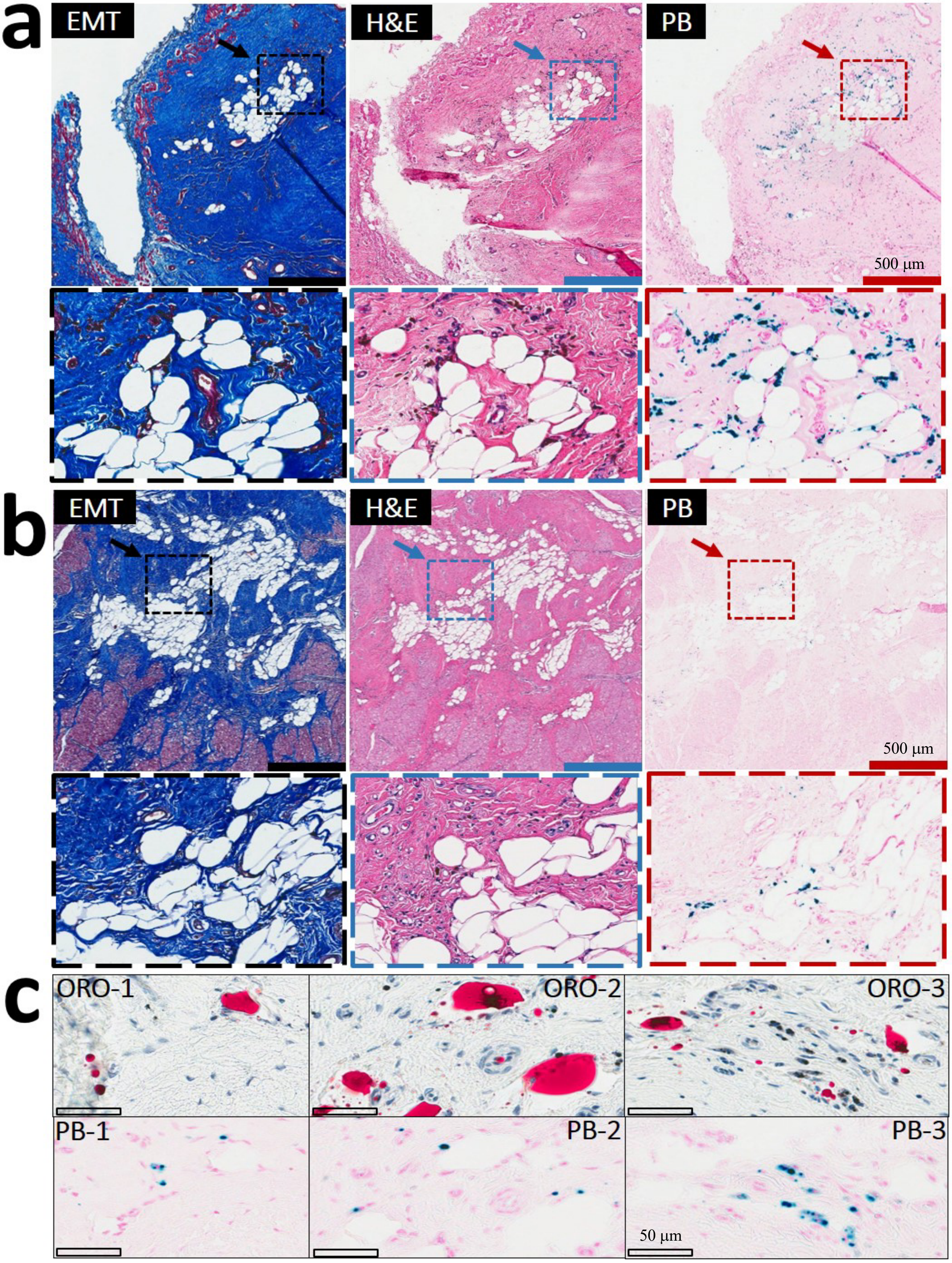

Supplementary Figure 6. Reperfusion hemorrhage-derived iron deposition carries a risk of lipomatous metaplasia in late chronic phase of post-MI scar. Serial *paraffin* sections from a 6-month-old hemorrhagic MI stained with elastin-modified Masson's trichrome (EMT), H&E and Prussian Blue (PB) stains are presented in Panels **a** and **b**. As evident in panel **b** (representing *subendocardial* region), individual foam cells were exclusively observed in the peri-infarct and border zones and exclusively co-localized with chronic iron deposits. Larger fat depots, as evident in *midmyocardial* region presented in panel **b**, typically penetrated scar tissue at its internal core. Notably, these larger foam cell clusters typically colocalized with iron deposits along the fat depot periphery while the core of the growing adipose tissue contained traces of iron deposits. Serial *frozen* sections from a dog with 6-month-old hemorrhagic MI stained with Oil-Red-O (ORO) and PB stains are presented in Panel **c**. Note that individual foam cells emerged exclusively at the confluence of iron deposits and lipid remnants from necrotic myocardium. Moreover, panel **c** supports the notion that even traces of iron deposits from reperfusion hemorrhage, if in contact with lipid remnants from necrotic myocardium, carry a risk for LM in old hemorrhagic scars. The number of samples per timepoint/animal group used is depicted in Supplementary Fig. 1.
Scale bar of images in panels **a** and **b** is 500 μm, while those in panel **c** is 50 μm.

9) **Supplementary Figure 7**

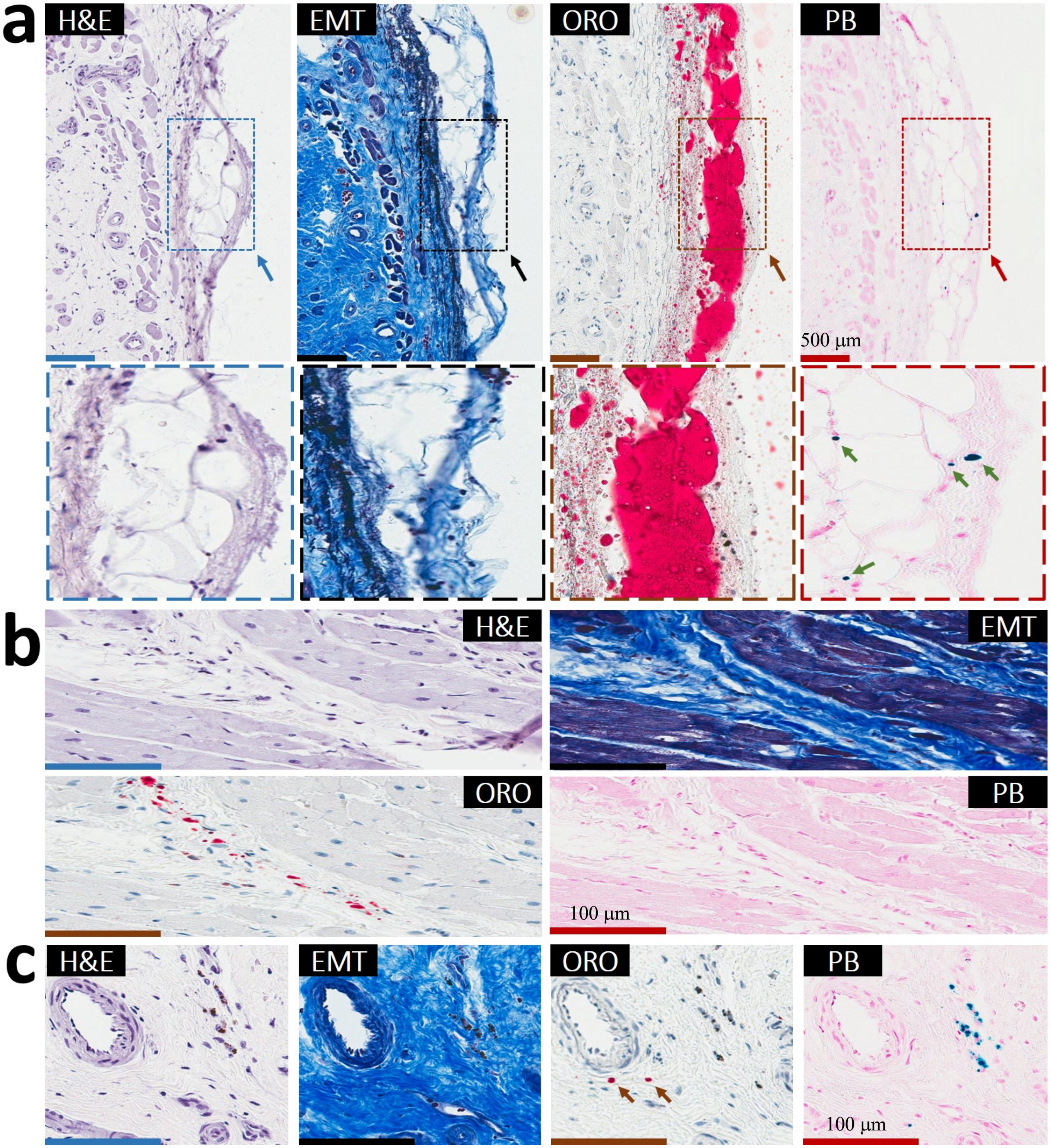

Supplementary Figure 7. Lipomatous metaplasia in the late chronic phase of hemorrhagic MI is unique to scarred regions with iron deposits and lipid remnants. Serial *frozen* sections from an 6-month-old hemorrhagic MI stained with H&E, elastin-modified Masson's trichrome (EMT), Oil-Red-O (ORO), and Prussian Blue (PB) stains are presented in panels **a** – **c**. A peri-infarct zone of sub-endocardium is presented in panel **c** (zoomed-in section of panel **a** (arrow) presented dotted rectangles). Note the extensive colocalization of iron deposits with mini clusters of fat. In contrast, regions without iron but with lipids (panel **b**) as well as regions with iron but no lipids (panel **c**) did not exhibit LM. Importantly, panel **c** also suggests that iron deposits and lipid remnants must be in the immediate proximity for LM to take place. Note the absence of foam cells/LM in this region with iron despite the fact that lipids are "close-by" but not in contact with iron remnants. The number of samples per timepoint/animal group used is depicted in Supplementary Fig. 1. Scale bar in panel **a** equals 500 µm while in panels **b** and **c** equals 100 µm.

10) Supplementary Figure 8

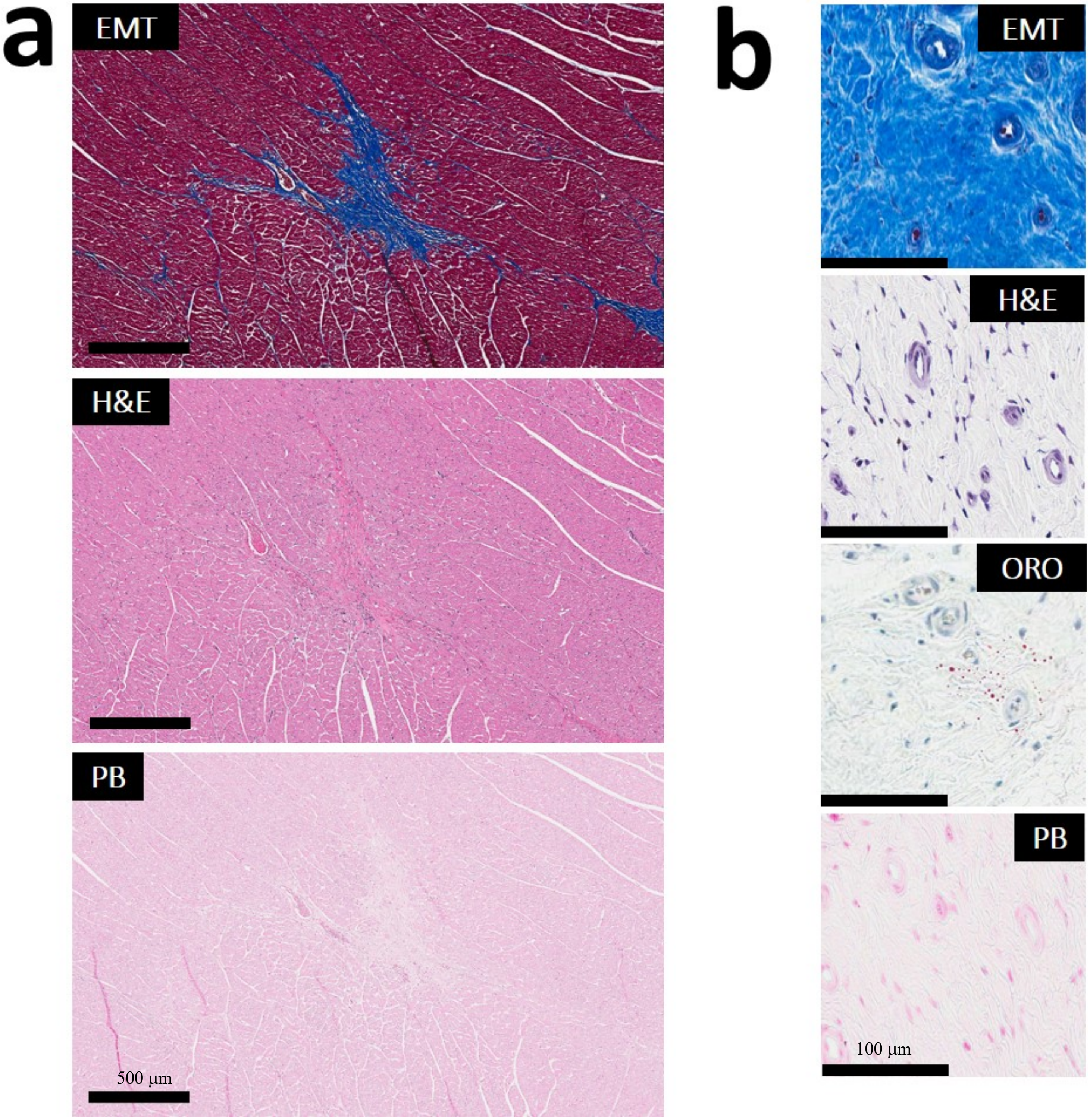

Supplementary Figure 8. Lipomatous metaplasia is also not evident in in the late chronic phase of non-hemorrhagic MI. Serial *paraffin* sections from an 6-month-old non-hemorrhagic MI stained with elastin-modified Masson's trichrome (EMT), H&E and Prussian Blue (PB) are presented in panel **a**. Old non-hemorrhagic MIs were consistently negative for iron, foam cells and lipomatous metaplasia. Given that the foam cell- and LM-negative scars consistently exhibited traces of ORO-stained lipid remnants in contiguous *frozen* sections (panel **b**) within old scars indicates that iron plays a critical role in driving lipomatous metaplasia and fat deposition in infarcted myocardium. The number of samples per timepoint/animal group used is depicted in Supplementary Fig. 1. Scale bar in panel **a** equals 500 μm, while in panel **b** equals 100 μm.

11) Supplementary Figure 9

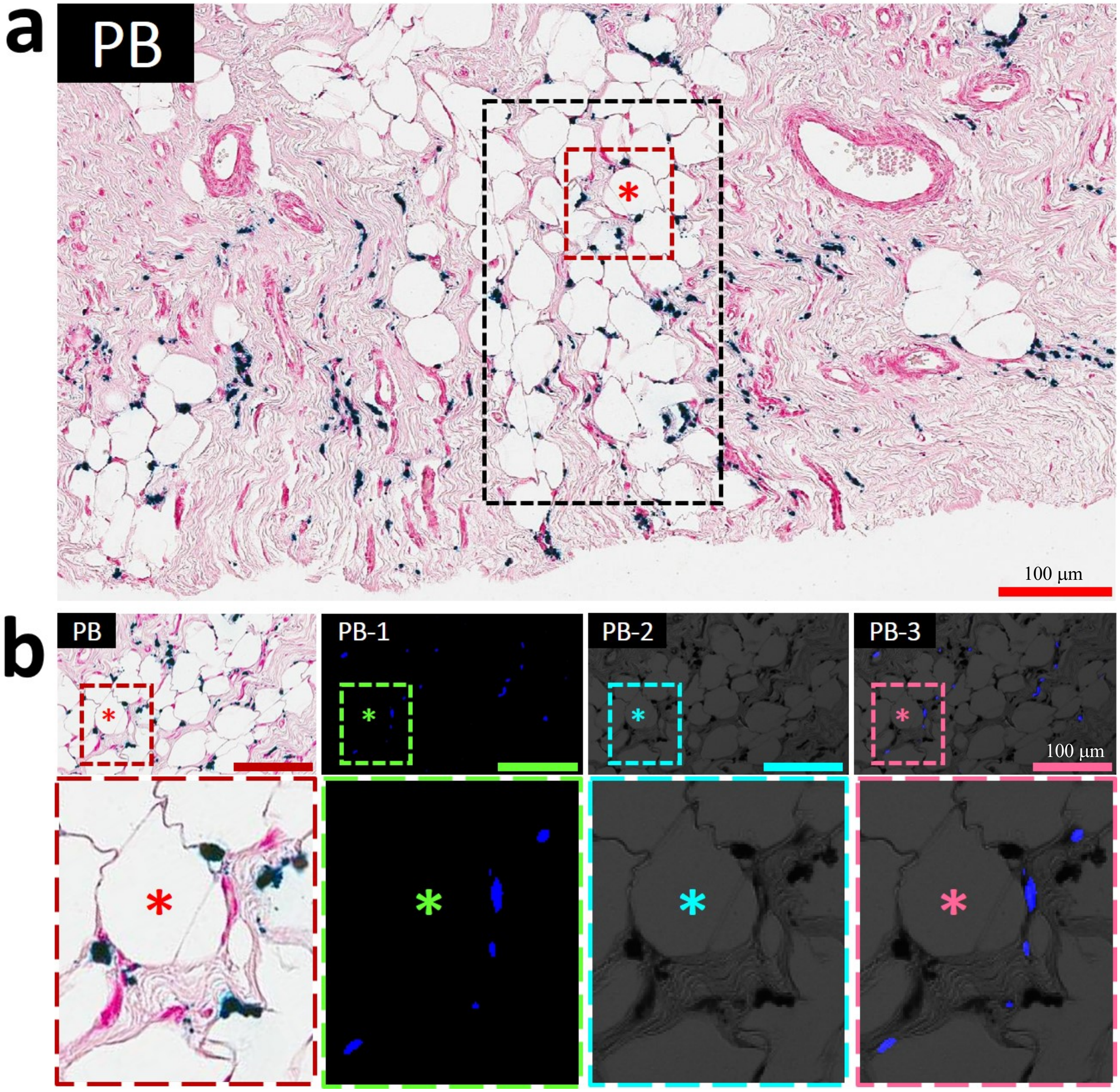

Supplementary Figure 9. Appearance of foam cells in the late chronic phase of hemorrhagic MI is accompanied by highly localized deposition of ceroid lipopigment in iron-laden Territories. Confocal microscopy evaluation of ceroid lipopigment in the Prussian blue-stained *paraffin* section (panel **a**) from a 6-month-old hemorrhagic MI is presented in panel **b**. Inset dotted border boxes containing the asterisks denote the corresponding zoomed-in regions directly below. **(PB-1)** Excitation wavelength: 405 nm and Emission wavelength: 428-496 nm. **(PB-2)** Differential Interference Contrast (DIC). **(PB-3)** Overlay. As evident in panel **a**, individual foam cells and mini clusters of foam cells observed in the peri-infarct and border zones exclusively colocalized with persistent iron deposits in old hemorrhagic scar. Moreover, these individual foam cells and mini clusters of foam cells also intensively colocalized with ceroid/autofluorescence in old hemorrhagic scars. The number of samples per timepoint/animal group used is depicted in Supplementary Fig. 1. Scale bar in panels **a** and **b** equals 100 μm.

12) Supplementary Figure 10

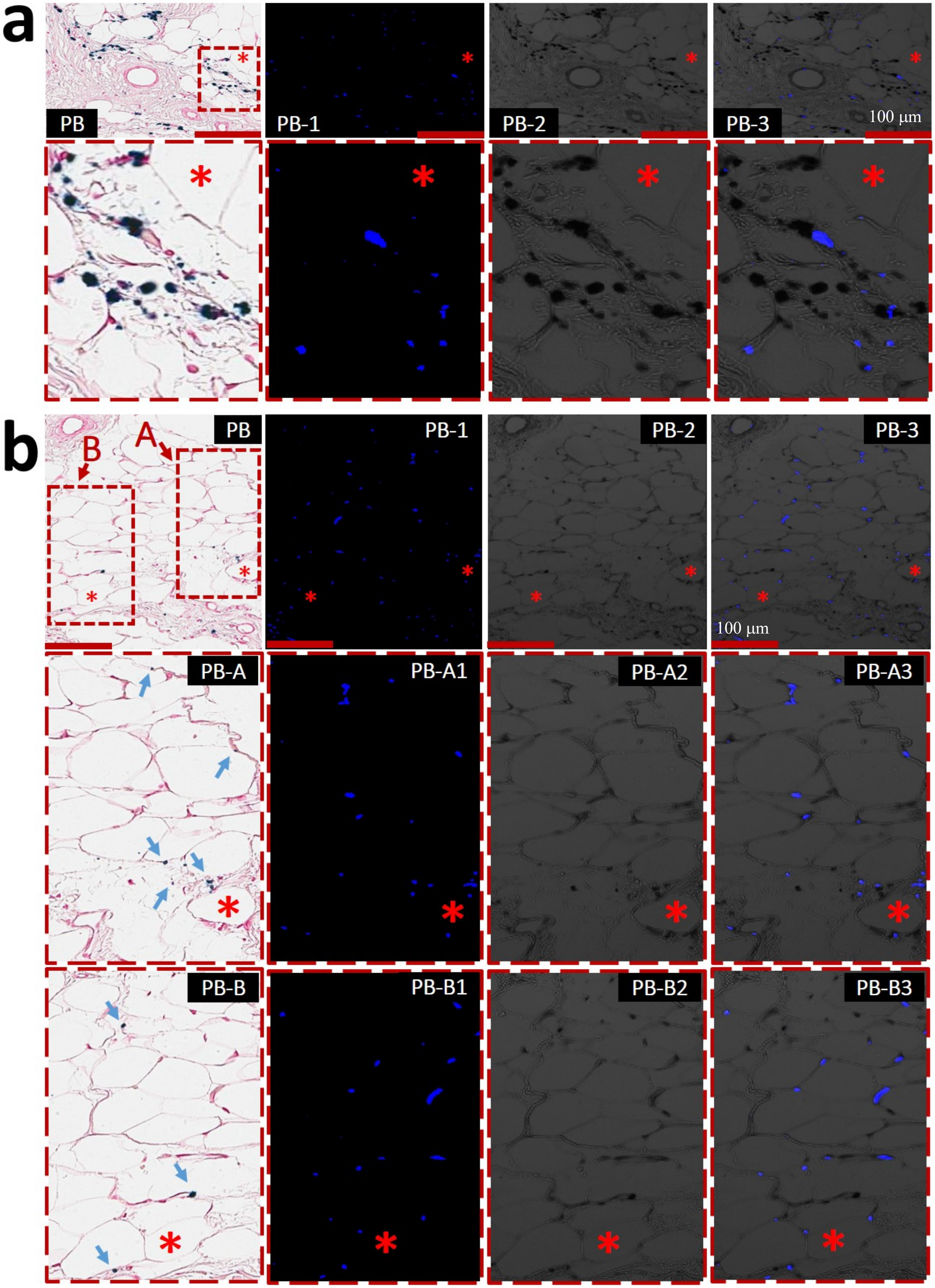

Supplementary Figure 10. Lipomatous metaplasia of hemorrhagic MI begins in the peri-infarct and border zones of infarcted myocardium and progressively invades the scar core. Confocal microscopy evaluation of ceroid lipopigment in the Prussian blue-stained *paraffin* sections from 6-month-old hemorrhagic MIs are presented in panels **a** and **b**. Inset boxes with asterisk correspond to zoomed-in regions. **(PB-1)** Excitation wavelength: 405 nm and Emission wavelength: 428-496 nm. **(PB-2)** Differential Interference Contrast (DIC), **(PB-3)** Overlay. Individual foam cells observed in the peri-infarct and border zones exclusively colocalized with persistent iron deposits in old hemorrhagic scar (panel **a**). Also, note that in larger fat depots, the autofluorescence of ceroid deposits colocalizing with iron remnants, was detected at both the fat depot periphery (iron-fat interface) (panel **a**) as well as in the core (panel **b**) of growing adipose tissue. Given that the ceroid-rich core of larger fat depots also contained traces of iron deposits (arrows), these findings suggest that fat deposition within MI begins in the scar periphery and progressively penetrates the MI core. The number of samples per timepoint/animal group used is depicted in Supplementary Fig. 1. Scale bar in panels **a** and **b** equals 100 μm.

13) Supplementary Figure 11

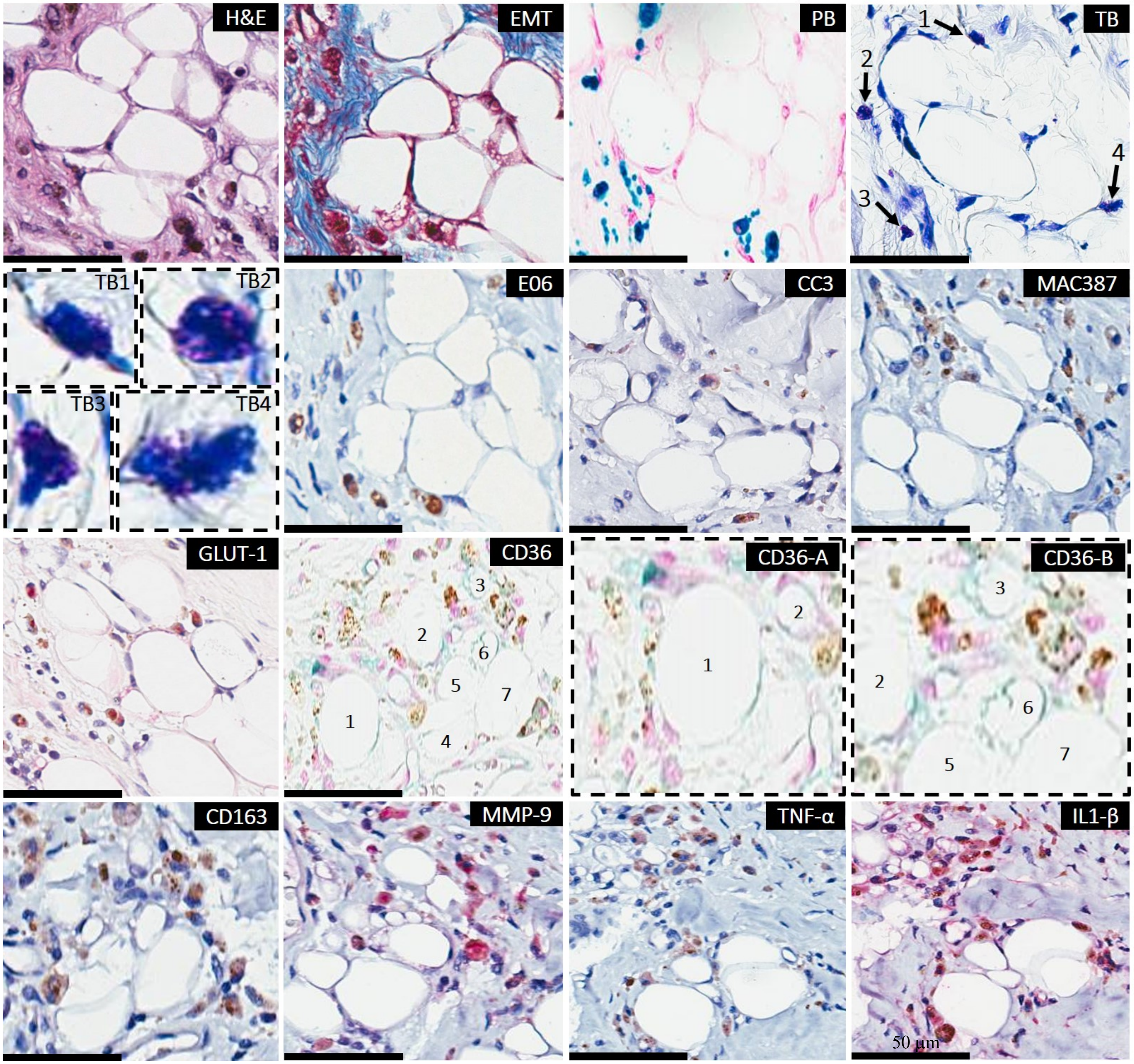

Supplementary Figure 11. Inability of iron-laden macrophages to switch from M1 to M2 phenotype is coincident with foam cell formation and lipomatous metaplasia in the late chronic phase of hemorrhagic MI. Representative serial *paraffin* histology sections from 6-month-old hemorrhagic MI were stained with H&E, elastin-modified Masson's trichrome (EMT), Prussian Blue (PB), Toluidine Blue (TB) as well as the anti-MAC387, anti-E06, anti-Cleaved Caspase 3 (CC3), anti-CD36, anti-CD163, anti-MMP-9, anti-TNF-α and anti-IL-1β antibodies. Persistent iron deposits (blue stain) co-localized with fat depots within old hemorrhagic scar. As observed in the early phase of chronic MI, increased homing of mast cells (TB, arrows) in iron-laden regions undergoing LM was also evident in the late chronic phase of MI. Note the individual degranulated mast cells in TB1-4. Increased generation of oxidized phospholipids in the "iron-mast cell-macrophage-fat territory" was evidenced by intense E06 staining (red staining). Positive immunohistochemical staining with anti-CC3 antibody confirmed the ongoing apoptosis of iron-laden macrophage-derived foam cells (red stain; arrows). New macrophages (MAC387+) were persistently recruited to the E06+ regions undergoing LM (brown staining). Retention of the glycolytic phenotype in macrophages undergoing foam cell transformation was confirmed by intense immunoreactivity for GLUT-1. Fat depots within "iron-mast cell territory" stained positive for CD36 (green) indicating macrophage-derived foam cell formation. Note the individual foam cells in CD36-A&B. CD163-positive macrophages (pink staining) co-localized with foam cells indicating iron-induced macrophage-to-foam cell transformation. Positive staining for MMP-9, TNF-α and IL-1β (all stained red) in the "iron-mast cell-macrophage-fat territory" indicates that hemorrhagic infarcts lead to prolonged mast cell-mediated inflammatory response culminating in fat deposition within post-MI scar. The number of samples per timepoint/animal group used is depicted in Supplementary Fig. 1. Scale bar equals 50 μm.

14) Supplementary Figure 12

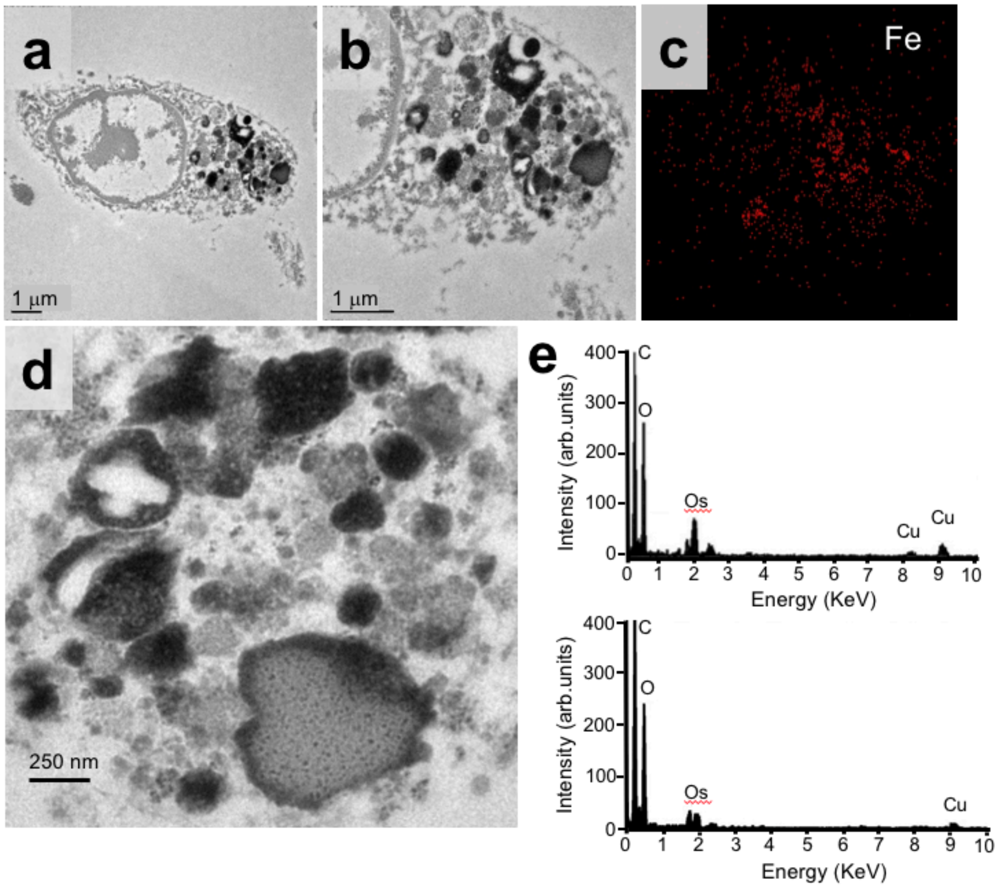

Supplementary Figure 12. Macrophages found in non-hemorrhagic MI territories lack iron and lipid globules. TEM image of a macrophage cell (**a**), and its zoomed-in region (**b**). Energy-dispersive X-ray spectroscopy (EDS) elemental map of Fe distribution within that area (**c**) showing a very low, background-level iron signal (red). Typical EDS spectra collected from the two region are shown in (**e**). Intracellular components in macrophages from hemorrhagic regions **(d)**, as compared to panels a, b and d in Fig. 7. The experiment was repeated independently three times with similar results.
