# Supplementary material for: Intramyocardial hemorrhage drives fatty degeneration of infarcted myocardium
Source: Nat Commun. 2022 Oct 27;13:6394. doi: 10.1038/s41467-022-33776-x (PMC9613644; doi:10.1038/s41467-022-33776-x)
Supplement: Supplementary file 3 — Reporting Summary [file 41467_2022_33776_MOESM3_ESM.pdf]

nature portfolio

Corresponding author(s): Rohan Dharmakumar

Last updated by author(s): 2022-9-30

Reporting Summary

Nature Portfolio wishes to improve the reproducibility of the work that we publish. This form provides structure for consistency and transparency in reporting. For further information on Nature Portfolio policies, see our Editorial Policies and the Editorial Policy Checklist.

Statistics

For all statistical analyses, confirm that the following items are present in the figure legend, table legend, main text, or Methods section.

n/a	Confirmed	
☐	☒	The exact sample size (*n*) for each experimental group/condition, given as a discrete number and unit of measurement
☐	☒	A statement on whether measurements were taken from distinct samples or whether the same sample was measured repeatedly
☐	☒	The statistical test(s) used AND whether they are one- or two-sided<br>*Only common tests should be described solely by name; describe more complex techniques in the Methods section.*
☐	☒	A description of all covariates tested
☐	☒	A description of any assumptions or corrections, such as tests of normality and adjustment for multiple comparisons
☐	☒	A full description of the statistical parameters including central tendency (e.g. means) or other basic estimates (e.g. regression coefficient) AND variation (e.g. standard deviation) or associated estimates of uncertainty (e.g. confidence intervals)
☐	☒	For null hypothesis testing, the test statistic (e.g. *F*, *t*, *r*) with confidence intervals, effect sizes, degrees of freedom and *P* value noted<br>*Give P values as exact values whenever suitable.*
☒	☐	For Bayesian analysis, information on the choice of priors and Markov chain Monte Carlo settings
☒	☐	For hierarchical and complex designs, identification of the appropriate level for tests and full reporting of outcomes
☐	☒	Estimates of effect sizes (e.g. Cohen's *d*, Pearson's *r*), indicating how they were calculated

Our web collection on statistics for biologists contains articles on many of the points above.

Software and code

Policy information about availability of computer code

Data collection	No Commercial, open source or custom computer code are used in this study.
Data analysis	N/A

For manuscripts utilizing custom algorithms or software that are central to the research but not yet described in published literature, software must be made available to editors and reviewers. We strongly encourage code deposition in a community repository (e.g. GitHub). See the Nature Portfolio guidelines for submitting code & software for further information.

Data

Policy information about availability of data

All manuscripts must include a data availability statement. This statement should provide the following information, where applicable:

- Accession codes, unique identifiers, or web links for publicly available datasets
- A description of any restrictions on data availability
- For clinical datasets or third party data, please ensure that the statement adheres to our policy

All data generated or analyzed during this study are included in this published article (and its supplementary information files).

Human research participants

Policy information about studies involving human research participants and Sex and Gender in Research.

Reporting on sex and gender	No human research subjects are used in this study.
Population characteristics	N/A
Recruitment	N/A
Ethics oversight	N/A

Note that full information on the approval of the study protocol must also be provided in the manuscript.

Field-specific reporting

Please select the one below that is the best fit for your research. If you are not sure, read the appropriate sections before making your selection.

[x] Life sciences [ ] Behavioural & social sciences [ ] Ecological, evolutionary & environmental sciences

For a reference copy of the document with all sections, see nature.com/documents/nr-reporting-summary-flat.pdf

Life sciences study design

All studies must disclose on these points even when the disclosure is negative.

Sample size	To estimate sample size, we initially conducted pilot studies with 3 animals and a power analysis was performed subsequently to determine the sample size that would ensure a power of at least 0.8. Shapiro-Wilk test and quantile-quantile plots were used to test the normality of the data.
Data exclusions	The study timeline, animal groups and terminal end points of the various investigations are outlined in Supplementary Figure 1. Briefly, animal numbers are narrowed down from n = 41 to n = 20 to ensure that animals from DFP+/IMH+ and DFP-/IMH+ groups were matched to MI size and iron content at Day 3 (i.e. prior to treatment)).Whenever possible (dotted lines), untreated animals from the Interventional cohort were shared with Observational cohort to ensure optimal use of animals.
Replication	Statistical analyses were performed using SPSS Statistics (version 21.0, IBM Corporation, Armonk, NY). To estimate sample size, we initially conducted pilot studies with 3 animals and a power analysis was performed subsequently to determine the sample size that would ensure a power of at least 0.8. Shapiro-Wilk test and quantile-quantile plots were used to test the normality of the data. Depending on the normality of the data, analysis of variance or Kruskal-Wallis test along with post-hoc analyses were used to compare measurements among the different groups. Bonferroni correction was used for multiple comparisons. The paired one-tailed Student's t-test was used for comparisons between two groups. Linear regression analyses were performed to evaluate the relation between relative R2* and relative PDFF at D3, Wk8 and M6. All data are shown as mean +/- SEM. Statistical significance was set at $p<0.05$.
Randomization	We tested our hypothesis in dogs subjected to ischemia followed by reperfusion in a series of studies comprising of an observational arm and an interventional arm. The observational arm was used to serially study the tissue specific changes in iron and fat over a 6-month period with cardiac MRI and histology following reperfused MIs with and without intra-myocardial hemorrhage. The interventional arm was used to investigate whether an intracellular iron chelator can disrupt the iron deposition, fat deposition and adverse remodeling in hemorrhagic MIs. The study timeline, animal groups and terminal end points of the various investigations are outlined in Supplementary Material, Supplementary Fig. 1.
Blinding	Throughout this study, to minimize bias, animals were randomized into groups by blinding the veterinary surgeons to hemorrhage status or treatment and CMR readers to treatment groups, and sequential assignment of animals to DFP treatment and control groups

Reporting for specific materials, systems and methods

We require information from authors about some types of materials, experimental systems and methods used in many studies. Here, indicate whether each material, system or method listed is relevant to your study. If you are not sure if a list item applies to your research, read the appropriate section before selecting a response.

Materials & experimental systems

n/a	Involved in the study
[ ]	[x] Antibodies
[x]	[ ] Eukaryotic cell lines
[x]	[ ] Palaeontology and archaeology
[ ]	[x] Animals and other organisms
[x]	[ ] Clinical data
[x]	[ ] Dual use research of concern

Methods

n/a	Involved in the study
[x]	[ ] ChIP-seq
[x]	[ ] Flow cytometry
[x]	[ ] MRI-based neuroimaging

Antibodies

Antibodies used

All antibodies and dilution used were included in the methods and supplementary material sections:
For specific targets, associated markers and antibodies used to probe myocardial tissue are shown in the following:
Newly recruited macrophages, MAC387 (Abcam ab22506)
Macrophage scavenger receptor, CD163 (Bioss, bs-2527R)
Proinflammatory cytokine, TNF-! (Abcam, ab6671)
Matrix degrading enzyme, MMP-9 (Abcam, ab38898)
Proinflammatory cytokine, IL-1" (Abcam, ab34837)
Glucose transporter, GLUT-1, (Biorbyt LLC, orb312259)
Oxidized Phospholipids E06, (Avanti Polar Lipids, 330001S)
Foam Cells CD36, (Sigma-Aldrich, AV48129)
Apoptosis Cleaved Caspase-3, (Cell Signaling, Asp175 (5A1))
For Western blot analyses, the following antibodies are used:
IL-1" (Abcam, ab9722), TNF-! (Santa Cruz, SC52746), HCP1 (Santa Cruz, SC393460), FTH1 (Cell Signaling, 3998), CD36 (Proteintech, 18836-1-AP), SR-AI (Abcam, ab183725), LOX-1 (Proteintech, 11837-1-AP), SR-BI (Abcam, ab52629), or GAPDH (Cell Signaling, 5174)

Validation

All antibodies used were described in the methods and supplementary material sections:
MAC387, (Abcam ab22506), https://www.abcam.com/s100a9--calprotectin-s100a8a9-complex-antibody-mac387-ab22506.html
CD163 (Bioss, bs-2527R), https://www.biossusa.com/products/bs-2527r
TNF-! (Abcam, ab6671), https://www.abcam.com/tnf-alpha-antibody-ab6671.html
MMP-9 (Abcam, ab38898), https://www.abcam.com/mmp9-antibody-ab38898.html
IL-1" (Abcam, ab34837), Description: Rabbit polyclonal to IL1 beta, Host species: Rabbit, Specificity: This antibody primarily recognizes the 17kDa mature form of IL1 beta. It also recognizes 10% of the precursor, 31kDa form of IL1 beta in cell lysates. The antibody does not react with IL1 alpha. Tested applications Suitable for: ELISA, RIA, WB, IP, IHC-P, IHC-Fr, Neutralising Species reactivity Reacts with: Dog, Human, Predicted to work with: Non human primates, Does not react with: Mouse, Rat, Rabbit. Immunogen: Recombinant IL1 beta (Human) produced in E.coli. The MW of the recombinant 153 amino acid IL1 beta was 17 kDa with the N terminal amino acid at position alanine 117. This cleavage site is generated by the IL1 beta converting enzyme (ICE, Capase 1). Positive control Supernatants or cell lysates from cultures of LPS stimulated human peripheral blood mononuclear cells. (PBMCs were stimulated for 24 hours with 1% human serum and 10ng/ml E.coli derived LPS.)
GLUT-1, (Biorbyt LLC, orb312259), https://www.biorbyt.com/glut1-antibody-orb312259.html
E06, (Avanti Polar Lipids, 330001S), https://www.sigmaaldrich.com/US/en/product/avanti/330001s
CD36, (Sigma-Aldrich, AV48129), https://www.sigmaaldrich.com/US/en/product/sigma/av48129
Apoptosis Cleaved Caspase-3, (Cell Signaling, Asp175 (5A1)), https://www.cellsignal.com/products/primary-antibodies/
cleavedcaspase-
3-asp175-5a1e-rabbit-mab/9664
IL-1" (Abcam, ab9722), https://www.abcam.com/il-1-beta-antibody-ab9722.html
TNF-! (Santa Cruz, SC52746), https://www.scbt.com/p/tnfalpha-antibody-52b83
HCP1 (Santa Cruz, SC393460), https://www.scbt.com/p/hcp1-antibody-b-4
FTH1 (Cell Signaling, 3998), https://www.cellsignal.com/products/primary-antibodies/fth1-antibody/3998
CD36 (Proteintech, 18836-1-AP), https://www.ptglab.com/products/CD36-Antibody-18836-1-AP.htm
SR-AI (Abcam, ab183725), https://www.abcam.com/srap-antibody-epr11776-ab183725.html
LOX-1 (Proteintech, 11837-1-AP), https://www.ptglab.com/products/OLR1-Antibody-11837-1-AP.htm
SR-BI (Abcam, ab52629), https://www.abcam.com/scavenging-receptor-sr-bi-antibody-ep1556y-ab52629.html
GAPDH (Cell Signaling, 5174), https://www.cellsignal.com/products/primary-antibodies/gapdh-d16h11-xp-rabbit-mab/5174

Animals and other research organisms

Policy information about studies involving animals; ARRIVE guidelines recommended for reporting animal research, and Sex and Gender in Research

Laboratory animals

A total of 84 mongrel dogs (20-25kg; female) were studied. The detail is list in supplementary figure 1.

Wild animals

N/A

Reporting on sex

To effectively test our hypothesis, a chronic study design was required, where we serially examined the temporal changes of fat

	within the MI zone in dogs with and without hemorrhage. Since the dogs were followed up until 6 months post-reperfusion, female dogs were preferred over the male counterparts because of their size, aggression, housing, docility as well as the need for daily treatment. We also did not want these differences to confound our study. Moreover, to the best of our knowledge, there are no reports of gender differences in iron within MI in human subjects. There is also no report of sex differences in lipomatous metaplasia in the context of myocardial infarction in humans. Although we did not study sex differences and how it may or may not contribute to lipomatous metaplasia, it is worth noting that we did control for other key factors (MI size and hemorrhage volume) in our interventional study using DFP.
Field-collected samples	This study does not involved any field-collected samples.
Ethics oversight	A total of 84 mongrel dogs (20-25kg; female) were studied according to the protocols approved by the Institutional Animal Care and Use Committees of Indiana University (protocol number: 21174), Cedars-Sinai Medical Center (protocol number: 7324) and Lawson Health Research Institute (protocol number: 2017-006). All procedures were under the guidelines stipulated by the NIH Guide for the Care and Use of Laboratory Animals or Canadian Council of Animal Care (CCAC). Consistent with the institution protocols, animal care and husbandry followed the NIH Guide for the Care and Use of Laboratory Animals.

Note that full information on the approval of the study protocol must also be provided in the manuscript.
